# The association between iliac vein compression degree and characteristics of first diagnosed left lower extremity deep vein thrombosis

**DOI:** 10.3389/fcvm.2022.1073586

**Published:** 2022-12-21

**Authors:** Yadong Shi, Yuan Yuan, Maofeng Gong, Haobo Su, Liang Chen, Hao Huang, Zhaoxuan Lu, Yangyi Zhou, Jianping Gu

**Affiliations:** The Department of Vascular and Interventional Radiology, Nanjing First Hospital, Nanjing Medical University, Nanjing, China

**Keywords:** deep vein thrombosis, common iliac vein compression, compression degree, thrombus burden, May-Thurner syndrome

## Abstract

**Objective:**

This study aimed to investigate the association between the left common iliac vein (CIV) compression degree and characteristics of first diagnosed left lower extremity deep vein thrombosis (DVT).

**Patients and methods:**

This was a single-center retrospective observational study. Between January 2015 and June 2022, first diagnosed left lower extremity DVT patients with enhanced computed tomography of lower extremities were included. Patient demographics, comorbidities, risk factors, DVT characteristics, and CIV compression degree were collected and analyzed. Logistic regressions were performed to evaluate the odds ratio (OR) with a 95% confidence interval (CI) of iliofemoral or mixed DVT vs. compression percentage. The association between compression percentage and iliofemoral or mixed DVT was evaluated on a continuous scale with restricted cubic splines (RCS). The association between compression percentage and thrombus burden was evaluated using the Spearman test.

**Results:**

A total of 196 (mean age, 61.8 ± 16.1 years; 86 males) patients were included. The median CIV compression percentage in iliofemoral or mixed DVT patients was significantly greater than in non-iliofemoral or non-mixed DVT, respectively (64.4 vs. 46.6%, *p* < 0.001; 67.8 vs. 54.8%, *p* = 0.004). CIV compression >50% was associated with significantly increased morbidity of iliofemoral DVT (adjusted OR, 2.96; 95% CI, 1.58–5.52; *p* = 0.001) or mixed DVT (adjusted OR, 2.39; 95% CI, 1.19–4.81; *p* = 0.014). RCS showed that a greater compression percentage was associated with a continuously increased OR of iliofemoral DVT (overall *p* = 0.003, non-linear *p* = 0.577) or mixed DVT (overall *p* = 0.020, non-linear *p* = 0.771). CIV compression percentage had a positive correlation with thrombus burden (*r*s = 0.284, *p* < 0.001).

**Conclusion:**

A greater left CIV compression percentage may be associated with increasing likelihood of more proximal location and severe clot extent in first diagnosed left lower extremity DVT.

## Introduction

Deep vein thrombosis (DVT) is a considerable global disease burden (1) that carries high morbidity and mortality (2, 3). The annual incidence of DVT was approximately 1 person per 1,000 populations (2–4), and the 30-day mortality was 4.6% (3). Although various risk factors of DVT have been identified (5), iliac vein compression syndrome as one of the risk factors may have been underappreciated (6). Cockett and Thomas first describe iliac vein compression syndrome (also known as May-Thurner syndrome or Cockett syndrome), indicating that the compressed left common iliac vein (CIV) by the right common iliac artery and lumbar vertebra may cause ipsilateral venous stasis and secondary thrombosis (7, 8).

Previous studies have demonstrated that increased CIV compression degree was associated with an increased risk of DVT (9, 10). However, studies regarding the relationship between CIV compression degree and DVT characteristics are rare. The previous data suggested that more than 50% of left iliofemoral DVT patients had significant left CIV compression (11, 12). Moreover, patients with significant CIV compression were more prone to iliofemoral DVT (13, 14). Based on the above evidence, we believe left CIV compression may play an important role in left iliofemoral DVT. In addition, we hypothesize that patients with greater CIV compression percentage are more vulnerable to more severe venous stasis during the thrombotic process, thus leading to greater thrombus extent and burden.

Unfortunately, no study has specifically evaluated the correlation between the DVT characteristics (including thrombus level, extent, and burden) and CIV compression percentage on a continuous scale. To explore whether a greater left CIV compression degree is associated with a more proximal or extended thrombus in the left lower extremity veins, the present study investigated the association between the risk of left iliofemoral DVT or mixed DVT and left CIV compression percentage on a continuous scale. Moreover, the correlation between thrombus burden and compression percentage was also evaluated. These results may be helpful in better understanding the impact of CIV compression on ipsilateral DVT.

## Patients and methods

### Study design

Between 1 January 2015 and 30 June 2022, consecutive first diagnosed lower extremity DVT patients were retrospectively reviewed. Two reviewers (YS and YY) independently searched the electronic medical record system and picture archiving and communication system to identify potential candidates. The inclusion criteria included first diagnosed DVT patients who underwent computed tomography venography (CTV) for the lower extremities. The exclusion criteria included bilateral DVT, right lower extremity DVT, DVT history, and unmeasurable CIV. Patient demographics (age and sex), onset time of DVT, comorbidities (hypertension, diabetes, coronary heart disease, cardiac insufficiency, neurovascular disease, and peripheral arterial disease), risk factors for VTE (immobility, thrombophilia, varicose veins, estrogen use, active cancer, and peripartum status) were collected. The D-dimer value was also collected and analyzed. The imaging information regarding DVT characteristics [inferior vena cava (IVC) involvement, DVT level, DVT extent, and thrombus burden] and CIV compression (CIV minimum diameter and compression percentage) were recorded. The study was conducted in accordance with the Declaration of Helsinki. The Institutional Review Board of the study hospital approved this study protocol, and informed consent was waived due to the retrospective study design.

### DVT and CIV compression degree assessment

Patients with DVT were initially diagnosed with ultrasound scanning. For patients planning to undergo endovascular treatment, The CTV examination, including IVC and lower extremity veins, was routinely performed within 48 h after diagnosis. The assessment of DVT levels (iliofemoral, femoropopliteal, and calf vein), DVT extent (mixed or non-mixed DVT), and thrombus burden were based on both ultrasound scan and CTV. Mixed DVT was defined as iliofemoral, femoropopliteal, and calf vein thrombosis simultaneously. The thrombus burden of proximal DVT was assessed using the Venous Registry Index recommended by the Society of Interventional Radiology (15). In this scoring system, the lower extremity venous system was divided into seven segments, including IVC, CIV, external iliac vein, common femoral vein, proximal half of femoral vein, distal half of femoral vein, and popliteal vein. Each segment can be scored 0 points (completely free of thrombus), 1 point (partially occluded), or 2 points (completely occluded), and the total score was calculated to evaluate thrombus burden.

**FIGURE 1:**
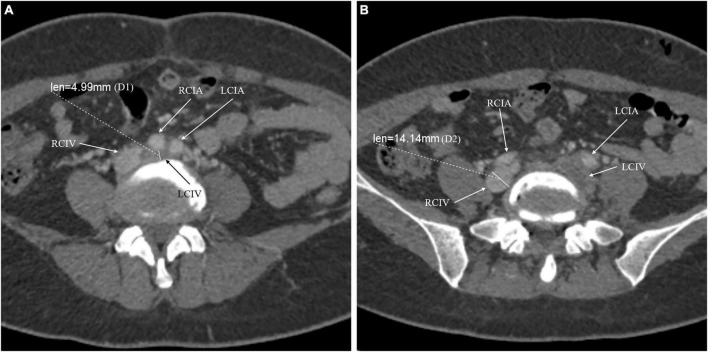
The compression percentage of the common iliac vein was calculated using the following formula: (1 – D1/D2) × 100%. D1 is the minimum diameter at the point of maximum compression **(A)**. Because the left common iliac vein was involved, D2 is the minimum diameter at the right common iliac vein caudal to the compression **(B)**. For instance, the compression percentage of LCIV for the presented patient was: (1 – 4.99/14.14) × 100% = 64.7%. RCIV, right common iliac vein; RCIA, right common iliac artery; LCIV, left common iliac vein; LCIA, left common iliac artery.

Left CIV compression was evaluated using quantitative and qualitative measures based on CTV. Adequate axial images were obtained, and the maximum CIV compression point was identified. The compression percentage was calculated using the formula (1 − D1/D2) × 100%, where D1 is the minimum diameter at the point of maximum compression, and D2 is the minimum diameter at the CIV caudal to the compression (16). For patients with left CIV thrombosis, we used the right distal CIV diameter as the reference denominator (Figure 1) (16). The qualitative estimate of CIV compression was performed according to the calculated compression percentage. The compression degree was classified into significant (compression percentage >50%) and non-significant (compression percentage ≤50%) (17).

Two board-certified radiologists blinded to the patient information independently assessed the CIV compression degree and thrombus burden. When there was disagreement between the reviewers, a third senior radiologist defined the final compression degree or thrombus burden.

### Statistical analysis

The distribution of continuous data was tested using the Kolmogorov–Smirnov test. Data with normal distribution were presented as mean ± SD. Data with asymmetric distribution were presented as the median and interquartile range (IQR). The violin plots were generated with GraphPad Prism (9.0v; GraphPad Software Inc., CA, USA). Student’s *t*-test or Mann–Whitney U test was used to compare the difference between continuous data, and the Chi-square test or Fisher’s exact test was used for count data. The association between the CIV compression percentage >50% (yes or no) and iliofemoral or mixed DVT was estimated by univariant and multivariant logistic regression models with an odds ratio (OR) and 95% confidence interval (CI).

To evaluate if greater compression percentage was associated with increasing greater OR of iliofemoral DVT or mixed DVT. The locally weighted scatterplot smoothing (LOWESS) plots were generated for exploratory analysis. Furthermore, the association between CIV compression percentage and iliofemoral DVT or mixed DVT was investigated using restricted cubic splines (RCS) based on adjusted logistic regression models. Knots are set at the 5th, 35th, 65th, and 95th percentiles of compression percentage. The LOWESS plots and logistic regression with RCS were performed by statistic software R (R Foundation for Statistical Computing, Vienna, Austria).^1^ The relationship between compression percentage and thrombus burden was evaluated using the Spearman test. A *p*-value < 0.05 was considered statistically significant.

## Results

### Patients

**FIGURE 2:**
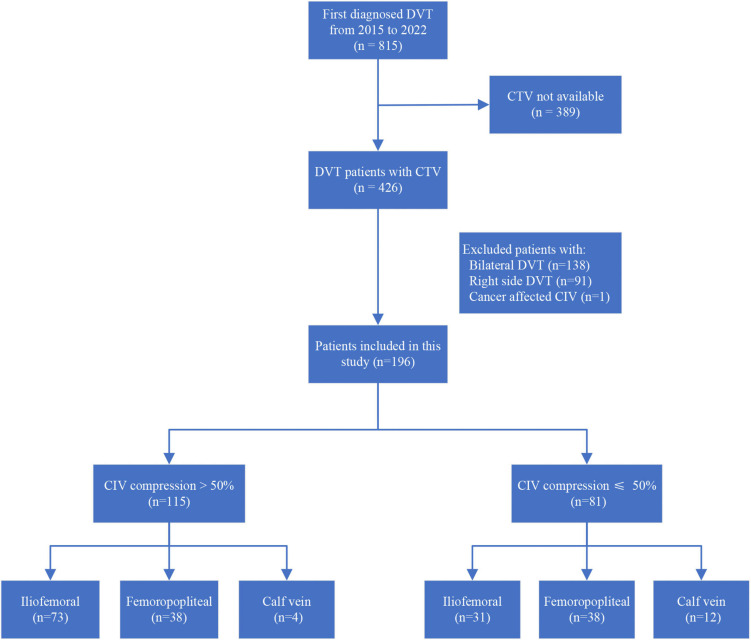
Flowchart of patient inclusion and DVT levels in patients with CIV compression percentage >50 or ≤50%. DVT, deep vein thrombosis; CTV, computed tomography venography; CIV, common iliac vein.

Between 1 January 2015 and 31 June 2022, 815 patients with first diagnosed DVT were identified in the study hospital. CTV was performed in 426 patients, whereas 230 patients were excluded for the following reasons: 138 patients had bilateral DVT, 91 patients had sole right side DVT, and one patient had cancer-involved CIV resulting in measurement impossible. Finally, 196 patients were included in the present study. The flow chart of patient inclusion is summarized in Figure 2.

The mean age of included patients was 61.8 ± 16.1 years, and 43.9% were male. The median onset time of DVT was 7.0 days (IQR: 3.0–14.0 days). More than 75% of patients had acute DVT, and the remaining patients had subacute DVT (Table 1). The leading comorbidities were hypertension and diabetes, presented in 39.8 and 16.3% of patients, respectively. In the present study, immobility was noted in 20.9% of included patients and was the major risk factor for DVT. Iliofemoral, femoropopliteal, and calf vein DVT were noted in 104 (53.1%), 76 (38.8%), and 16 (8.2%) patients. Iliofemoral DVT was more often found in patients with significant CIV compression (63.5 vs. 38.3%, *p* < 0.001). CTV demonstrated that all identified CIV compressions were caused by adjacent arteries. The median CIV minimum diameter and compression percentage were 5.0 mm (IQR, 3.3–7.6 mm) and 59.4% (IQR, 38.4–73.7%), respectively.

**TABLE 1:** Demographics, comorbidities, risk factors, and CIV compression degree in patients with and without iliofemoral DVT.

Variable	All patients (*n* = 196)	Iliofemoral DVT (*n* = 104)	Non-iliofemoral DVT (*n* = 92)	*p*-Value
**Demographics**				
Age (year)	61.8 ± 16.1	62.6 ± 16.5	60.8 ± 15.7	0.449
Male	86 (43.9%)	43 (41.3%)	43 (46.7%)	0.448
Median onset time (day) (IQR)	7.0 (3.0–14.0)	5.0 (2.0–14.0)	7.0 (3.0–13.0)	0.241
**DVT chronicity**				
Acute	149 (76.0%)	79 (76.0%)	70 (76.1%)	0.984
Median D-dimer (μg/ml) (IQR)	6.3 (3.4–11.7)	7.5 (4.7–16.3)	4.8 (2.5–9.4)	<0.001
**Comorbidities**				
Hypertension	78 (39.8%)	40 (38.5%)	38 (41.3%)	0.685
Diabetes	32 (16.3%)	15 (14.4%)	17 (18.5%)	0.443
Coronary heart disease	16 (8.2%)	6 (5.8%)	10 (10.9%)	0.193
Neurovascular disease	27 (13.8%)	16 (15.4%)	11 (12.0%)	0.487
Peripheral arterial diseases	21 (10.7%)	13 (12.5%)	8 (8.7%)	0.390
**Risk factors**				
Immobility	41 (20.9%)	23 (22.1%)	18 (19.6%)	0.661
Varicose veins	11 (5.6%)	6 (5.7%)	5 (5.4%)	0.919
Estrogen use	3 (1.5%)	2 (1.9%)	1 (1.1%)	1.000
Cancer	18 (9.2%)	14 (13.5%)	4 (4.3%)	0.027
Peripartum	7 (3.6%)	6 (5.7%)	1 (1.1%)	0.168
Concomitant symptomatic PE	31 (15.8%)	8 (7.7%)	23 (25.0%)	0.001
IVC involvement	25 (12.8%)	18 (17.3%)	7 (7.6%)	0.042
**CIV compression degree**				
Median CIV minimum diameter (mm) (IQR)	5.0 (3.3–7.6)	4.1 (3.0–6.3)	6.6 (3.9–8.4)	<0.001
Median CIV compression percentage (IQR)	59.4% (38.4–73.7%)	64.4% (44.5–77.2%)	46.6% (33.3–66.7%)	<0.001

Data are presented as *n* (%) or mean ± SD unless stated otherwise. CIV, common iliac vein; DVT, deep vein thrombosis; IQR, interquartile range; PE, pulmonary embolism; IVC, inferior vena cava.

### The association between CIV compression and iliofemoral DVT

**FIGURE 3:**
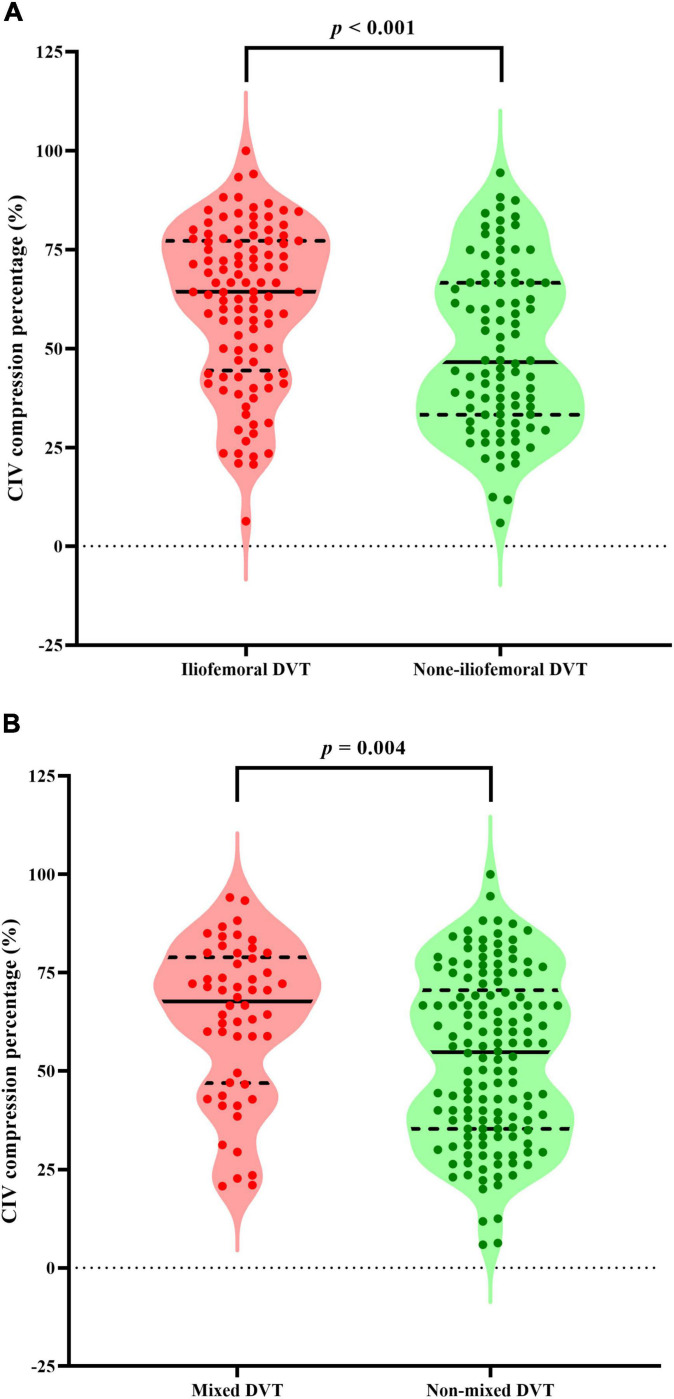
Violin plots presenting all points and the median value (solid lines) with interquartile range (dashed lines) of CIV compression percentage for patients with iliofemoral DVT **(A)** or mixed DVT **(B)**. Mann–Whitney U tests show that patients with iliofemoral DVT or mixed DVT had significantly greater median compression percentage than those without iliofemoral DVT or mixed DVT, respectively (*p* < 0.001 and *p* = 0.004, respectively). CIV, common iliac vein; DVT, deep vein thrombosis.

The patients with iliofemoral DVT had significantly smaller median CIV minimum diameter (4.1 vs. 6.6 mm, *p* < 0.001) and greater median compression percentage (64.4 vs. 46.6%, *p* < 0.001) (Figure 3A) than those without iliofemoral DVT. The univariant analysis found that the D-dimer level and cancer were significantly different in patients with and without iliofemoral DVT (Table 1).

The logistic regression showed that the likelihood of iliofemoral DVT in patients with compression percentage >50% was higher than in those with ≤50% (OR, 2.80; 95% CI, 1.56–5.04; *p* = 0.001). The correlation remained after applying adjusted Model 2 (OR, 2.84; 95% CI, 1.55–5.18; *p* = 0.001) or Model 3 (OR, 2.96; 95% CI, 1.58–5.52; *p* = 0.001) (Table 2). Model 2 was adjusted by age and sex. Model 3 was adjusted by age, sex, cancer, and D-dimer.

**TABLE 2:** Logistic regression model for the association between iliofemoral DVT and CIV compression percentage.

	Model 1^a^			Model 2^b^			Model 3^c^		
	OR	95% CI	*p*-Value	OR	95% CI	*p*-Value	OR	95% CI	*p*-Value
**CIV compression percentage**									
≤50%	1.00			1.00			1.00		
>50%	2.80	1.56–5.04	0.001	2.84	1.55–5.18	0.001	2.96	1.58–5.52	0.001
Age				1.01	0.99–1.03	0.374	1.01	0.99–1.03	0.368
Male				1.00	0.55–1.81	0.992	1.01	0.54–1.87	0.983
Cancer							3.54	1.06–11.78	0.040
D-dimer							1.04	1.01–1.07	0.015

^a^Model was not adjusted.

^b^Model adjusted for age and sex.

^c^Model adjusted for age, sex, cancer, and D-dimer level. DVT, deep vein thrombosis;

CIV, common iliac vein; OR, odds ratio; CI, confidence interval.

**FIGURE 4:**
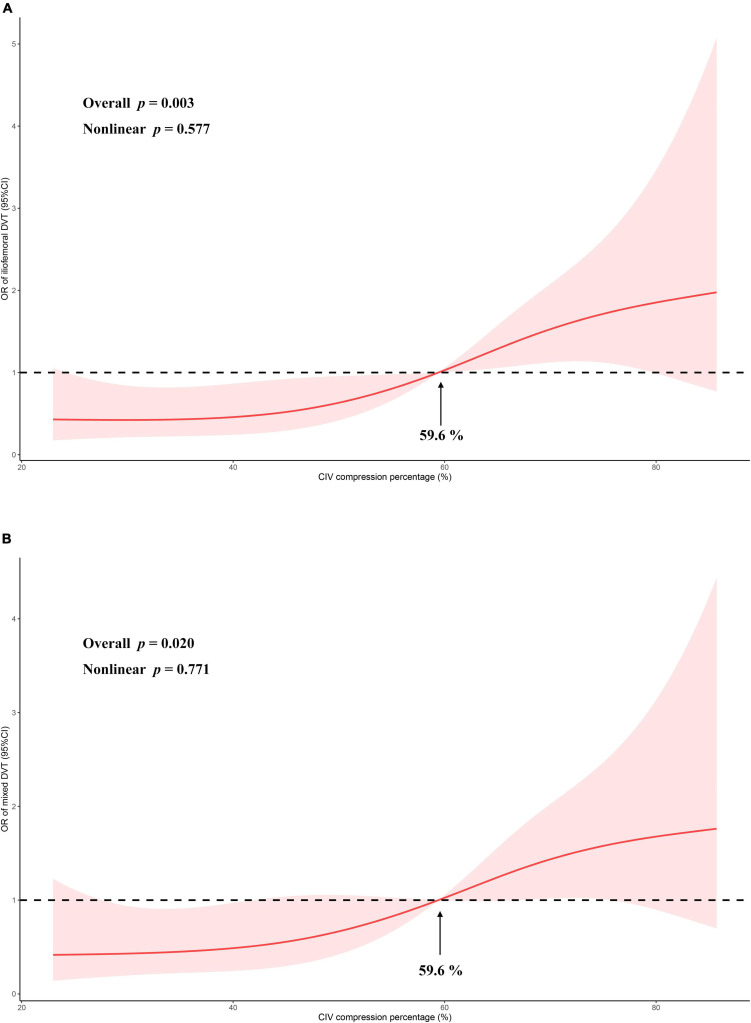
The odds ratio of CIV compression percentage vs. iliofemoral DVT **(A)** or mixed DVT **(B)** is modeled using logistic regressions with restricted cubic splines with 95% confidence limits (red ribbon). Analysis between compression percentage and iliofemoral DVT was adjusted for age, sex, D-dimer level, and cancer. Analysis between compression percentage and mixed DVT was adjusted for age and sex. The black dashed lines indicate reference lines for no association at an odds ratio of 1. Knots are set at the 5th, 35th, 65th, and 95th percentiles of CIV compression percentage. OR, odds ratio; CIV, common iliac vein.

Restricted cubic splines based on the logistic regression model adjusted by age, sex, cancer, and D-dimer showed that the increasing compression percentage was associated with increasing OR of iliofemoral DVT (overall *p* = 0.003, non-linear *p* = 0.577) (Figure 4A).

### The association between CIV compression and mixed DVT

The differences between patients with and without mixed DVT are presented in Table 3. The patients with mixed DVT had significantly smaller median CIV minimum diameter (4.1 vs. 5.4 mm, *p* = 0.013) and greater median compression percentage (67.8 vs. 54.8%, *p* = 0.004) (Figure 3B) than those without mixed DVT. However, no other significant difference was found between the groups.

**TABLE 3:** Demographics, comorbidities, risk factors, and CIV compression degree in patients with and without mixed DVT.

Variable	Mixed DVT (*n* = 54)	Non-mixed DVT (*n* = 142)	*p*-Value
**Demographics**			
Age (year)	63.5 ± 15.7	61.1 ± 16.3	0.359
Male	24 (44.4%)	62 (43.4%)	0.448
Median onset time (day) (IQR)	7.0 (2.8–14.3)	7.0 (3.0–13.0)	0.989
Acute DVT	41 (75.9%)	108 (76.1%)	0.985
Median D-dimer (μg/ml) (IQR)	7.2 (4.4–15.5)	4.8 (2.5–9.4)	0.122
**Comorbidities**			
Hypertension	22 (40.7%)	56 (39.4%)	0.868
Diabetes	7 (13.0%)	25 (17.6%)	0.432
Coronary heart disease	2 (3.7%)	14 (9.9%)	0.265
Neurovascular disease	7 (13.0%)	20 (14.1%)	0.839
Peripheral arterial diseases	7 (13.0%)	13 (9.2%)	0.431
**Risk factors**			
Immobility	15 (27.8%)	26 (18.3%)	0.145
Varicose veins	3 (5.6%)	8 (5.6%)	1.000
Estrogen use	0	3 (2.1%)	0.563
Cancer	4 (7.4%)	14 (9.9%)	0.799
Peripartum	3 (5.6%)	4 (2.8%)	0.623
**CIV compression degree**			
Median CIV minimum diameter (mm) (IQR)	4.1 (2.9–6.1)	5.4 (2.5–8.1)	0.013
Median CIV compression percentage (IQR)	67.8% (47.0–79.0%)	54.8% (35.3–70.6%)	0.004

Data are presented as *n* (%) or mean ± SD unless stated otherwise. CIV, common iliac vein; DVT, deep vein thrombosis; IQR, interquartile range; PE, pulmonary embolism; IVC, inferior vena cava.

The logistic regression showed that the likelihood of mixed DVT in patients with compression >50% was higher than in those with ≤50% (OR, 2.26; 95% CI, 1.14–4.46; *p* = 0.019). The correlation remained after applying Model 2 adjusted by age and sex (OR, 2.39; 95% CI, 1.19–4.81; *p* = 0.014) (Table 4).

**TABLE 4:** Logistic regression model for the association between mixed DVT and CIV compression percentage.

	Model 1^a^			Model 2^b^		
	OR	95% CI	*p*-Value	OR	95% CI	*p-*Value
**CIV compression percentage**						
≤50%	1.00			1.00		
>50%	2.26	1.14–4.46	0.019	2.39	1.19–4.81	0.014
Age				1.01	0.99–1.03	0.300
Male				1.25	0.65–2.42	0.505

^a^Model was not adjusted.

^b^Model adjusted for age and sex.

DVT, deep vein thrombosis; CIV, common iliac vein; OR, odds ratio; CI, confidence interval; IVCF, inferior vena cava filter.

Restricted cubic splines based on the logistic regression model adjusted by age and sex showed that the increasing compression percentage was associated with increasing OR of mixed DVT (overall *p* = 0.020, non-linear *p* = 0.771) (Figure 4B).

### The impact of CIV compression percentage on thrombus burden and thrombus extension to the IVC

**FIGURE 5:**
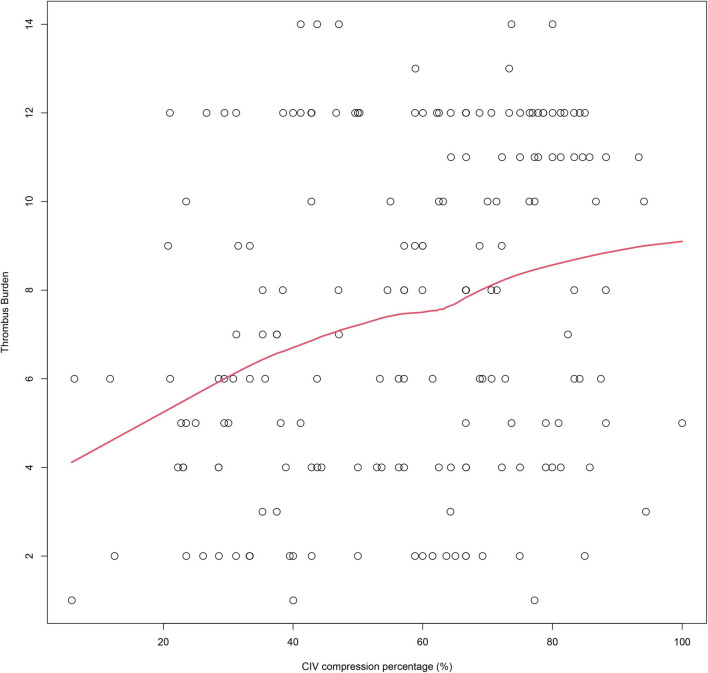
The LOWESS plots show the association between CIV compression percentage and thrombus burden. LOWESS, locally weighted scatterplot smoothing; CIV, common iliac vein.

Among included patients, 180 had proximal DVT, and the median thrombus burden for these patients was 7 points (IQR, 4–11 points). The LOWESS plots suggest that the increasing CIV compression percentage was associated with increasing thrombus burden (Figure 5). Moreover, the Spearman test showed that CIV compression percentage positively correlated with thrombus burden in proximal DVT (*r*s = 0.244, *p* = 0.001). The median thrombus burden in patients with CIV compression >50% was significantly greater than those with ≤50% (8.0 vs. 6.0, *p* = 0.020).

Thrombus extension to the IVC was noted in 12.8% (25/196) of the patients. The incidence of IVC involvement was not significantly different in patients with CIV compression >50% and those with ≤50% (11.3 vs. 14.8%, *p* = 0.468).

## Discussion

Significant CIV compression as a considerable risk for ipsilateral DVT has been well recognized (5, 8, 9). However, the impact of CIV compression degree on first diagnosed DVT characteristics has not been well elucidated, and only limited evidence was available. In a retrospective study that included 75 patients with DVT, Chan et al. (14) reported that 35% of patients with CIV stenosis (vein diameter <4 mm) had CIV thrombosis, which was significantly higher than patients without stenosis. In another study including 112 DVT patients, Jin et al. (13) reported that patients with May-Thurner syndrome are more prone to have iliofemoral DVT than those without May-Thurner syndrome (38.0 vs. 6.1%, *p* < 0.001). Moreover, previous studies reported that venous spurs were presented in one-half to two-thirds of left iliofemoral DVTs (6), and more than three-fourths of left iliofemoral DVT patients had severe CIV compression (compression percentage > 70%) (12). In the present study, 70.9% (73/104) of patients with iliofemoral DVT had significant CIV compression, and iliofemoral DVT was more often found in patients with significant CIV compression (63.5 vs. 38.3%, *p* < 0.001). These results were comparable to previous studies.

Furthermore, the present study further evaluated the association between CIV compression degree and DVT characteristics and revealed several important findings. First, multivariant regression revealed that DVT patients with CIV compression had a higher likelihood of iliofemoral DVT (OR, 2.96) or mixed DVT (OR, 2.39). Second, the RCS based on adjusted logistic regression revealed that an increasing CIV compression percentage was associated with a consistent increasing OR of iliofemoral DVT or mixed DVT. Third, the LOWESS plots and Spearman test showed that the greater CIV compression percentage positively correlated with the greater thrombus burden.

The main pathophysiological factors in DVT were increased procoagulant activity in the blood, vein wall damage, and impaired venous flow (Virchow’s triad) (5). Extrinsic CIV compression may cause venous stasis and repetitive endothelial injury at the site of compression, which predisposes to venous thrombosis *in situ* (5). Clinically reported DVT due to CIV compression accounts for only 2 to 5%, whereas the true proportion may have been substantially underappreciated (6). Additionally, CIV compression may become a physical barrier during thrombus migration (14), resulting in thrombus accumulation in the CIV. The present study found that the likelihood of iliofemoral DVT in patients with significant CIV compression was almost threefold compared with those without compression. The increasing CIV compression was also associated with increasing OR of iliofemoral DVT. These findings highlight the influence of CIV compression degree on iliofemoral DVT. Moreover, the increasing CIV compression degree may lead to more severe venous stasis during the thrombotic process, which may accelerate thrombus propagation. Unfortunately, the association between CIV compression percentage and thrombus extension has not been studied. The present study revealed that compression percentage positively correlated with thrombus burden and mixed DVT risk. Moreover, the patients with significant compression had greater thrombus burden than those without significant compression. These findings suggest that greater CIV compression might be associated not only with thrombosis but also with thrombus propagation. Such findings expand the knowledge regarding the impact of CIV compression on DVT.

The association between CIV compression and thrombus extension to the IVC has not been well documented. Previous studies found that ipsilateral significant CIV compression reduced the incidence of pulmonary embolism in DVT patients (13, 14, 18). A reasonable explanation for this phenomenon is that the significantly compressed CIV, with or without subsequent iliac vein thrombosis, may serve as a physical barrier that avoids thrombus migration or extension (14, 18, 19). However, Shi et al. (20) claimed that patients with May-Thurner syndrome are at risk for thrombus extension to the IVC. The present study evaluated the association between IVC involvement and CIV compression, and we found that the incidence of IVC involvement in patients with significant CIV compression was slightly lower than in those without compression, whereas the difference was not significant (*p* = 0.468). This insignificance may be attributed to the limited event number, and future studies are required to answer this issue.

Despite adequate anticoagulant therapy, up to 50% of patients with extensive DVT will develop post-thrombotic syndrome (21), which could impair the patient’s quality of life (22). Previous studies have found that the adjunctive iliac vein stenting for DVT patients with iliac vein compression syndrome was associated with improved venous patency and quality of life (23, 24). The present study implicates that potential CIV compression should be considered and evaluated in patients with extensive DVT, and subsequent early thrombus removal and correction of possible CIV compression should be considered in these patients to avoid post-thrombotic syndrome (5, 25).

There are some important limitations to the present study. First, the present study was a single-center retrospective study with a relatively small case number. Limited by the study design, selection bias might have influenced the reliability. The requirement of CTV may have biased the study toward a selection of patients with more severe DVT. Thus, the results should be interpreted with caution. Second, the thrombus burden evaluated by the scoring system was specifically designed for proximal DVT, and the assessment of thrombus burden in calf veins was impossible (26). Third, anticoagulant therapy was initiated before the CTV examination in some patients, and this confounder may have somewhat influenced the final results. Fourth, intravascular ultrasound, which was not performed in this study, may be better for locating and grading the stenosis (27). However, intravascular ultrasound is invasive, and a previous study suggested CTV had comparable power for evaluating CIV compression (16). Despite the limitations mentioned above, this is still the first study investigating the association between DVT characteristics and CIV compression on a continuous scale.

## Conclusion

In conclusion, an increasing CIV compression percentage may be associated with a consistently increasing OR of iliofemoral DVT and mixed DVT in first diagnosed left lower extremity DVT. The greater CIV compression percentage also positively correlated with the greater thrombus burden. These findings expanded the knowledge regarding the role of CIV compression in DVT. We may also benefit from the clinical implications of these findings and possibly improve the management of DVT.

## Data availability statement

The original contributions presented in this study are included in the article/supplementary material, further inquiries can be directed to the corresponding author.

## Ethics statement

The studies involving human participants were reviewed and approved by the Institutional Review Board of Nanjing First Hospital. Written informed consent for participation was not required for this study in accordance with the national legislation and the institutional requirements.

## Author contributions

JG and YS: study design. YS, HS, and YY: data collection. MG, HH, and ZL: data analysis. YS and LC: writing. All authors contributed to the article and approved the submitted version.

## Footnotes

1
http://www.R-project.org
